# Catastrophic health spending in Europe: equity and policy implications of different calculation methods

**DOI:** 10.2471/BLT.18.209031

**Published:** 2018-06-04

**Authors:** Jonathan Cylus, Sarah Thomson, Tamás Evetovits

**Affiliations:** a European Observatory on Health Systems and Policies, London School of Economics and Political Science, SHF 2.03, Houghton Street, WC2A 2AE London, England.; b WHO Barcelona Office for Health Systems Strengthening, World Health Organization Regional Office for Europe, Barcelona, Spain.

## Abstract

**Objective:**

To investigate the equity and policy implications of different methods to calculate catastrophic health spending.

**Methods:**

We used routinely collected data from recent household budget surveys in 14 European countries. We calculated the incidence of catastrophic health spending and its distribution across consumption quintiles using four methods. We compared the budget share method, which is used to monitor universal health coverage (UHC) in the sustainable development goals (SDGs), with three other well-established methods: actual food spending; partial normative food spending; and normative spending on food, housing and utilities.

**Findings:**

Country estimates of the incidence of catastrophic health spending were generally similar using the normative spending on food, housing and utilities method and the budget share method at the 10% threshold of a household’s ability to pay. The former method found that catastrophic spending was concentrated in the poorest quintile in all countries, whereas with the budget share method catastrophic spending was largely experienced by richer households. This is because the threshold for catastrophic health spending in the budget share method is the same for all households, while the other methods generated effective thresholds that varied across households. The normative spending on food, housing and utilities method was the only one that produced an effective threshold that rose smoothly with total household expenditure.

**Conclusion:**

The budget share method used in the SDGs overestimates financial hardship among rich households and underestimates hardship among poor households. This raises concerns about the ability of the SDG process to generate appropriate guidance for policy on UHC.

## Résumé

**Objectif:**

Examiner les incidences sur l'équité et les politiques de différentes méthodes utilisées pour calculer les dépenses de santé exorbitantes.

**Méthodes:**

Nous avons utilisé des données systématiquement recueillies dans le cadre d'enquêtes récentes sur le budget des ménages menées dans 14 pays européens. Nous avons calculé l'incidence des dépenses de santé exorbitantes et leur répartition par quintile de consommation à l'aide de quatre méthodes. Nous avons comparé la méthode de la part budgétaire, qui est utilisée pour suivre la couverture sanitaire universelle dans le cadre des objectifs de développement durable (ODD), à trois autres méthodes classiques: les dépenses alimentaires réelles; les dépenses normatives partielles relatives à l'alimentation; et les dépenses normatives relatives à l'alimentation, au logement et aux services collectifs.

**Résultats:**

Les estimations par pays de l'incidence des dépenses de santé exorbitantes étaient généralement similaires avec la méthode des dépenses normatives relatives à l'alimentation, au logement et aux services collectifs et la méthode de la part budgétaire au seuil de 10 % de la capacité de payer d'un ménage. La première méthode a révélé que les dépenses exorbitantes se concentraient sur le quintile le plus pauvre dans tous les pays, tandis que selon la méthode de la part budgétaire, les dépenses exorbitantes étaient en grande partie subies par des ménages plus riches. Cela s'explique par le fait que le seuil pour les dépenses de santé exorbitantes avec la méthode de la part budgétaire est le même pour tous les ménages, alors que les autres méthodes généraient des seuils efficaces qui variaient selon les ménages. La méthode des dépenses normatives relatives à l'alimentation, au logement et aux services collectifs était la seule à produire un seuil efficace qui augmentait de façon régulière avec les dépenses totales des ménages.

**Conclusion:**

La méthode de la part budgétaire utilisée dans le cadre des ODD surestime les difficultés financières des ménages riches et sous-estime les difficultés rencontrées par les ménages pauvres. Cela suscite des inquiétudes quant à la capacité du processus des ODD à fournir des orientations appropriées pour les politiques relatives à la couverture sanitaire universelle.****

## Resumen

**Objetivo:**

investigar la equidad y las implicaciones políticas de los diferentes métodos para calcular el gasto sanitario catastrófico.

**Métodos:**

se han usado datos recopilados de forma rutinaria a partir de encuestas recientes sobre presupuestos domésticos en 14 países europeos. Se calculó la incidencia del gasto sanitario catastrófico y su distribución en los quintiles de consumo empleando cuatro métodos. Se comparó el método de asignación presupuestaria, que se emplea para monitorizar la cobertura sanitaria universal (CSU) dentro de los objetivos de desarrollo sostenible (ODS) con otros tres métodos perfectamente establecidos: gasto alimentario real, gasto alimentario parcial normativo y gasto normativo en alimentación, alojamiento y servicios.

**Resultados:**

las estimaciones de los países sobre la incidencia del gasto sanitario catastrófico eran en general similares usando el método del gasto normativo en alimentos, alojamiento y servicios y el método de asignación presupuestaria en el umbral del 10% de la capacidad de un hogar para pagar. El primer método descubrió que el gasto catastrófico se concentraba en el quintil más pobre de todos los países, mientras que el método de asignación presupuestaria se experimentaba en gran medida en hogares con mayor poder adquisitivo. Esto se debe a que el umbral del gasto sanitario catastrófico dentro del método de asignación presupuestaria es el mismo para todos los hogares, mientras que los demás métodos generaban umbrales efectivos que variaban conforme a los hogares. El método del gasto normativo en alimentos, alojamiento y servicios era el único que creaba un umbral efectivo que aumentaba suavemente con el gasto doméstico total.

**Conclusión:**

el método de asignación presupuestaria que se usa en los ODS sobreestima las dificultades económicas entre los hogares ricos y subestima las dificultades entre los hogares pobres. Esto desata preocupaciones acerca de la capacidad del proceso de los ODS de generar una guía adecuada para la política de la CSU.

## ملخص

**الهدف:**

التحقيق في المساواة وانعكاسات السياسات للطرق المختلفة لحساب الإنفاق الكارثي على الصحة.

**الطريقة:**

استخدمنا البيانات التي يتم جمعها بشكل روتيني من الاستبيانات الحديثة للميزانيات الأسرية في 14 دولة أوروبية. وقمنا بحساب معدل الإنفاق الكارثي على الصحة وتوزيعه عبر الفئات الخمسية للاستهلاك باستخدام أربع طرق. ثم قمنا بمقارنة طريقة حصة الميزانية، والتي يتم استخدامها لرصد التغطية الصحية الشاملة (UHC) في أهداف التنمية المستدامة (SDGs)، مع ثلاث طرق أخرى مستقرة: الإنفاق الفعلي على الغذاء؛ والإنفاق المعياري الجزئي على الغذاء؛ والإنفاق المعياري على الغذاء، والإسكان والمرافق.

**النتائج:**

كانت التقديرات القطرية المتعلقة بمعدل الإنفاق الكارثي على الصحة متشابهة بصفة عامة، وذلك باستخدام طريقة الإنفاق المعياري على الغذاء والسكن والمرافق، وطريقة حصة الميزانية عند عتبة 10٪ من قدرة الأسرة على الصرف. ووجدت الطريقة الأولى أن الإنفاق الكارثي كان يتركز في الفئات الخمسية الأكثر فقراً في كل البلدان، بينما كان الإنفاق الكارثي لطريقة حصة الميزانية هو الأكثر انتشاراً بين الأسر الأكثر ثراء. ويرجع ذلك إلى أن عتبة الإنفاق الكارثي على الصحة في طريقة حصة الميزانية هي نفسها بالنسبة لكل الأسر، بينما أنتجت الأساليب الأخرى عتبات فعالة تتنوع بين الأسر. وكانت طريقة الإنفاق المعياري على الغذاء والسكن والمرافق، هي الأسلوب الوحيد الذي أنتج عتبة فعالة ارتفعت بسلاسة مع إجمالي الإنفاق الأسري.

**الاستنتاج:**

إن طريقة حصة الميزانية المستخدمة في أهداف التنمية المستدامة (SDG) تبالغ في تقدير الصعوبات المالية بين الأسر الغنية، وتقلل من شأن الصعوبات بين الأسر الفقيرة. ويثير ذلك المخاوف بشأن قدرة عملية أهداف التنمية المستدامة على وضع إرشادات ملائمة لسياسة التغطية الصحية الشاملة. ****

## 摘要

**目的:**

调查灾害性卫生支出的不同计算方法的公平性和政策含义。

**方法:**

我们使用了来自 14 个欧洲国家的近期家庭预算调查中的常规数据。我们使用四种方法计算了灾害性卫生支出的发生率及其在消费五分位数中的分布情况。我们对比了预算份额法和其他三种成熟方法，前者是用于监控可持续发展目标 (SDG) 中的全民健康覆盖 (UHC)，后者则包括实际食品支出，部分规范性食品支出，以及在食品、住房和公共事业方面的规范性支出。

**结果:**

在食品、住房和公共事业方面的规范性支出与预算份额法估算的国家灾害性卫生支出的发生率基本相似，占家庭支付能力阈值的 10%。前一种方法发现所有国家的灾害性支出均集中于最贫困的五分之一地区，而预算份额法中灾害性支出很大程度上是集中于富裕家庭。这是因为预算份额法中所有家庭灾害性支出的阈值都一样，而由有效阈值产生的其他方法在各个家庭之间有所差异。在食品、住房和公共事业方面的规范性支出是唯一能够产生有效阈值且随着整个家庭支出稳定提升。

**结论:**

可持续发展目标 (SDG) 中使用的预算份额法高估了富裕家庭的经济困难程度，同时低估了贫困家庭的经济困难程度。这引发了对可持续发展目标 (SDG) 进程为全民健康覆盖 (UHC) 制定适当指导政策能力的担忧。

## Резюме

**Цель:**

Изучить справедливость и выводы для экономической политики с использованием различных методов расчета критически высоких расходов на здравоохранение.

**Методы:**

Авторы использовали данные, собираемые на регулярной основе в ходе недавних обследований бюджетов домашних хозяйств в 14 европейских странах. Были произведены расчет частоты критически высоких расходов на здравоохранение и ее распределение по квинтилям затрат с использованием четырех методов. Авторы сравнили метод распределения бюджета по статьям расходов, который используется для мониторинга всеобщего охвата медико-санитарными услугами в рамках целей устойчивого развития (ЦУР), с тремя другими общепринятыми методами, учитывающими фактические расходы на питание, частичные нормативные расходы на питание и нормативные расходы на питание и жилищно-коммунальные услуги.

**Результаты:**

Оценки по стране в отношении критически высоких расходов на здравоохранение, как правило, были схожи при использовании метода, учитывающего нормативные расходы на питание и жилищно-коммунальные услуги, и метода распределения бюджета по статьям расходов при пороге 10% от платежеспособности домашнего хозяйства. При использовании предыдущего метода было установлено, что критически высокие расходы были сосредоточены в беднейшем квинтиле во всех странах, тогда как при использовании метода распределения бюджета по статьям расходов критически высокие расходы в основном были связаны с более богатыми домашними хозяйствами. Это связано с тем, что порог критически высоких расходов на здравоохранение при использовании метода распределения бюджета по статьям расходов одинаков для всех домашних хозяйств, тогда как другие методы создают эффективные пороговые значения, которые варьируются в разных домашних хозяйствах. Метод, учитывающий нормативные расходы на питание и жилищно-коммунальные услуги, был единственным, который создал эффективный порог, плавно растущий вместе с ростом общих расходов домашних хозяйств.

**Вывод:**

Метод распределения бюджета по статьям расходов, используемый в рамках ЦУР, дает завышенную оценку финансовых трудностей среди богатых домашних хозяйств и заниженную оценку среди бедных. Это вызывает сомнения относительно способности процесса в рамках ЦУР создавать условия для составления соответствующих рекомендаций для политики в области всеобщего охвата медико-санитарными услугами.****

## Introduction

Catastrophic health spending is an established indicator of financial protection used to monitor global progress towards universal health coverage (UHC),^1^^–^^7^ as set out in *Transforming our world: the 2030 agenda for sustainable development*.^8^ It is defined as health spending that exceeds a predefined percentage or threshold of a household’s ability to pay for health care. However, ability to pay can be interpreted in different ways, leading to measurement differences.

The simplest approach assumes that a household’s entire budget is available for health-care spending. This is known as the budget share method, because it considers health spending in relation to total household expenditure or, less frequently, to income. Other approaches reject this assumption on the grounds that households must first meet basic needs, such as food and shelter, before covering health-care expenses. These methods consider health spending in relation to household expenditure minus an amount representing spending on basic needs. The remaining balance is referred to as a household’s capacity-to-pay for health care.

For the sustainable development goals (SDGs), catastrophic spending on health is monitored using the budget share method. SDG indicator 3.8.2 defines the incidence of catastrophic health spending as “the proportion of the population with large household expenditure on health as a share of total household expenditure or income.”^9^ Two thresholds are used to define large: 10% and 25%. While the budget share method has the virtue of simplicity, it has drawbacks.^6^

To illustrate these drawbacks, we compared the budget share method with three other methods widely applied at the global or the regional level to calculate the incidence of catastrophic health spending. These methods are: (i) the actual food spending method used by the Pan American Health Organization and others;^3^^,^^10^ (ii) the partial normative food spending method^11^ used by the World Health Organization (WHO) and others;^1^^–^^3^ and (iii) the normative spending on food, housing and utilities method, developed by the WHO Regional Office for Europe.^12^ The term normative is used for methods that reflect a judgement on how much households have to spend to meet basic needs. While earlier analyses have found some variability across the different methods in terms of overall levels of incidence of catastrophic health spending,^3^^,^^13^ we aimed to document the extent of variation among rich and poor households, and the reasons for this variation.

## Methods

### Measures

All the methods select out-of-pocket payments as the numerator for calculating the incidence of catastrophic health spending. This is because other sources of health spending (e.g. insurance premiums, contributions or taxes) are explicitly designed to protect against the financial risk associated with ill health, via pre-payment and risk pooling. Out-of-pocket payments include formal and informal payments made by households at the point of using any health goods or service offered by any type of provider, net of reimbursement from a third party.

We can argue that the basis for the denominator, income or consumption expenditure, should be determined by whether we think it is right for people to draw on their savings, sell assets or borrow to pay for health care. If we choose income, we assume people have no other resources available to pay for health care, which we know is not the case. Neither income nor consumption expenditure perfectly captures a household’s available resources. The research, however, consistently favours consumption over income for two reasons: it is deemed to be a better indicator of welfare, especially in poorer countries, and it is easier to measure accurately.^14^^,^^15^

The methods commonly used to measure catastrophic health spending are distinguished primarily by how they define a household’s ability to pay for health care. In the budget share method, the denominator is total household expenditure (or sometimes income), which assumes that all of a household’s resources are available for spending on health. In contrast, the other three methods assume that households have to meet basic needs before they can spend on health. These so-called capacity-to-pay methods define ability to pay for health care as total household expenditure minus an amount corresponding to spending on basic needs. Each method defines this amount differently (Table 1).

**Table 1:** Comparison of four methods used to calculate the incidence of catastrophic health spending

Method	Numerator	Basis for the denominator	Denominator	Basic needs used to calculate household ability (or capacity) to pay for health care	Thresholds typically used to signify catastrophic spending	Use in global or regional universal health coverage monitoring
Budget share	Out-of-pocket payments	Household total expenditure if available, otherwise income	Household total expenditure if available, otherwise total income	None	10% and 25%	SDGs; WHO; World Bank
Actual food spending	Out-of-pocket payments	Household total expenditure	Household total expenditure minus actual food spending	Household actual food spending	25% and 40%	PAHO; World Bank
Partial normative food spending	Out-of-pocket payments	Household total expenditure	Household spending minus a standard amount representing subsistence food spending. Except for households which are already below the subsistence level; in that case use household total expenditure minus actual food spending	Average food spending per (equivalent)^a^ person among households whose food share of total spending is between 45th and 55th percentiles	40%	WHO
Normative spending on food, housing and utilities	Out-of-pocket payments	Household total expenditure	Household total expenditure minus a standard amount representing subsistence spending on food, rent^b^ and utilities (water, electricity, gas and other fuels); applied to all households so that some very poor households may have negative capacity-to-pay	Food, rent and utilities spending per (equivalent)^a^ person (for households that spend on these items) between the 25th and 35th percentiles of total spending per (equivalent)^a^ person (using the average for this percentile range)	40%	WHO Regional Office for Europe

PAHO: Pan American Health Organization; SDGs: sustainable development goals; WHO: World Health Organization.

^a^ To adjust for household composition we used Organisation for Economic Co-operation and Development equivalence scales.^16^

^b^ Rent payments are considered as consumption expenditure in household budget surveys, but mortgage payments are regarded as investments and usually not collected. To address this anomaly, many countries (e.g. all European Union countries except Czechia and the United Kingdom of Great Britain and Northern Ireland) impute household rent for non-renters; however, the imputation methods vary substantially across countries.^17^ Since these imputed rent levels are sensitive to the choice of imputation method, the normative spending method excludes imputed rent from total household consumption expenditure to enhance cross-country comparability. Adjusting for rent payment among households living in rented accommodation therefore becomes important since without doing so, renters would systematically appear wealthier than otherwise identical owner-occupied households.

Notes: Spending refers to household consumption expenditure, which is the sum of the monetary value of all items consumed by the household during a given period and the imputed value of items that are not purchased but procured for consumption in other ways (for example, food reared or grown by the household). Threshold refers to the share of total household expenditure or capacity-to-pay which, when exceeded, indicates a household has experienced catastrophic health spending.

There are also differences in the threshold used to identify catastrophic spending. While any threshold is arbitrary, the budget share method uses both 10% and 25% in the SDGs. The partial normative food-spending method and normative spending on food, housing and utilities method primarily use 40%, whereas the actual food-spending method commonly uses both 25% and 40%. To facilitate comparison across methods, while remaining consistent in how these methods have been applied previously, we used 40% thresholds for all methods except the budget share method.

The official SDG indicator reports population-weighted incidence, while other methods often report household-weighted incidence. To ensure comparability across methods, we present all results at the household level, as this is the unit of data collection and analysis in household budget surveys. The SDG indicator also constructs consumption quintiles based on consumption expenditure per person. This method may be inappropriate since it ignores economies of scale within a household, underestimating welfare in households with many children, for example.^18^ For comparability, we constructed all quintiles based on per equivalent person consumption expenditure levels using Organisation for Economic Co-operation and Development^16^ equivalence scales (1.0 for the first adult, 0.7 for subsequent adults and 0.5 for children younger than 13 years).

### Data sources

We used routinely collected household budget survey data for 14 countries: Austria, Czechia, Estonia, France, Georgia, Germany, Hungary, Kyrgyzstan, Latvia, Lithuania, the Republic of Moldova, Poland, Sweden and the United Kingdom of Great Britain and Northern Ireland. These countries were chosen to reflect diversity in economic development and health-system design across the WHO European Region (Table 2). Data for the most recent year available were obtained from national statistics offices by local experts as part of a study commissioned by WHO Regional Office for Europe. All categorize household spending using the United Nations’ Classification of Individual Consumption According to Purpose.^20^

**Table 2:** Selected characteristics of countries in the study of catastrophic health spending in Europe

Country	Survey year	Population in millions	Gross domestic product in constant (2010) PPP per capita	General government expenditure in constant (2010) PPP per capita	Current health expenditure per capita in PPP	Compulsory financing arrangements as % of current health expenditure	Type of purchasing arrangement for publicly financed health care
Austria	2015	8.6	43 066	22 250	5 138	76	Regional non-competing health insurance funds
Czechia	2012	10.5	27 905	12 412	2 043	84	Competing health insurance funds
Estonia	2015	1.3	25 988	10 490	1 887	76	Single health insurance fund
France	2011	65.0	36 801	20 579	4 040	78	Non-competing health insurance funds
Georgia	2015	4.0	8 327	2 445	718	39	Single purchasing agency
Germany	2013	80.6	41 675	18 634	4 965	84	Competing health insurance funds
Hungary	2014	9.9	23 117	11 331	1 820	67	Single health insurance fund
Kyrgyzstan	2014	5.8	3 150	1 082	282	46	Single health insurance fund
Latvia	2013	2.0	14 879	7 648	1 219	60	Single purchasing agency
Lithuania	2012	3.0	22 859	8 253	1 542	67	Single health insurance fund
Republic of Moldova	2013	3.6	4 449	1 716	485	51	Single health insurance fund
Poland	2014	38.0	23 580	9 964	1 608	71	Regional non-competing health insurance funds
Sweden	2012	9.5	42 185	21 824	4 911	84	Regional non-competing purchasing agencies
United Kingdom	2014	64.4	37 661	16 464	4 009	80	Regional non-competing purchasing agencies

PPP: purchasing power parity.

Source: WHO Global Health Expenditure Database.^19^

### Data analysis

We conducted a retrospective observational study where we calculated the incidence of catastrophic health spending for each method for each country. We examined the distribution of catastrophic out-of-pocket payments across consumption quintiles, as a proxy for wealth, to identify inequalities in financial protection. The data were analysed using Stata, version 12 (Stata Corp., College Station, United States of America).

## Results

**Fig. 1:**
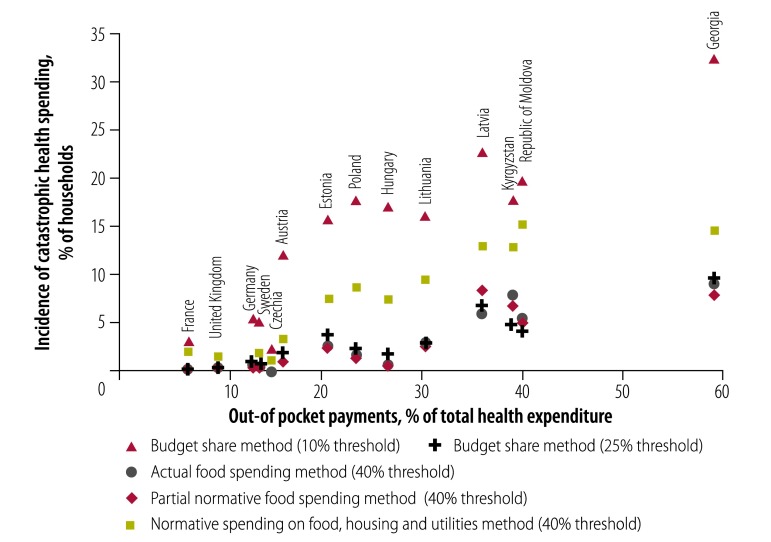
Incidence of catastrophic health spending, using different methods of calculation, and out-of-pocket payments as a share of total health expenditure in 14 European countries

Notes: The number of households surveyed in countries (survey year) were: Austria (2015): 7162; WHO Regional Office for Europe (2012): 2896; Estonia (2015): 33 989; France (2011): 3395; Georgia (2015): 9983; Germany (2013): 10 999; Hungary (2014): 6872; Kyrgyzstan (2014): 4665; Latvia (2013): 6931; Lithuania (2012): 3931; Republic of Moldova (2013): 5082; Poland (2014): 37 214; Sweden (2012): 1520; United Kingdom of Great of Britain and Northern Ireland (2014): 5733. Data on out-of-pocket payments as a share of total spending on health are from the WHO Global Health Expenditure Database.^19^

Using the budget share method, the 10% threshold of a household’s ability to pay resulted in the highest incidence of catastrophic health spending in all countries. The share of households affected ranged from just over 2% in Czechia to nearly 33% in Georgia (Fig. 1). The same method applied with a threshold of 25% resulted in a much lower incidence of catastrophic health spending across countries, with a range of 0% in Czechia to 9% in Georgia, but with some changes in country rankings. Results for the actual food spending and the partial normative food-spending methods were very similar to those of the budget share method (25% threshold). The normative spending on food, housing and utilities method resulted in levels of catastrophic spending that were considerably lower than the budget share method (10% threshold) but higher than the other methods, with a range of 1% in Czechia to 15% in the Republic of Moldova.

Fig. 1 also compared the incidence of catastrophic health spending for each method to out-of-pocket payments as a share of total health expenditure, a more readily available ratio often used as a proxy for financial hardship. Three methods, budget share (25% threshold), actual food spending and partial normative food methods, showed very little variation in incidence of catastrophic health spending as the out-of-pocket share of total health expenditure rose across countries. In the Republic of Moldova, for example, where out-of-pocket expenditure comprised 41% of total spending in 2013, these three methods found that catastrophic spending affected fewer than 5% of households. Results for the budget share (10% threshold) and the normative spending on food, housing and utilities methods more visibly reflected cross-country differences in health system financing: countries with greater reliance on out-of-pocket payments had a progressively higher incidence of catastrophic spending.

**Fig. 2:**
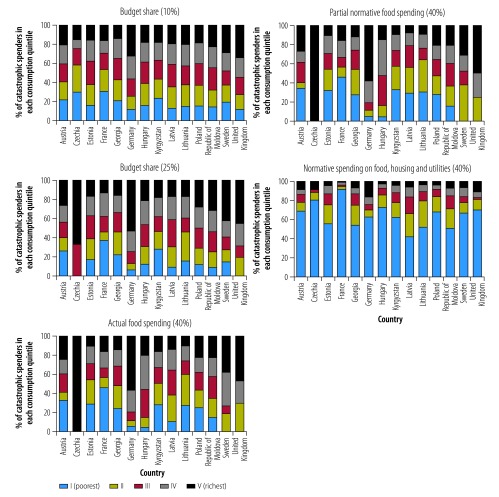
Incidence of catastrophic health spending by consumption quintile in 14 European countries, using different methods of calculation

Notes: Survey years and samples sizes are listed in the footnote to Fig. 1. Threshold refers to the share of total household expenditure or capacity-to-pay which, when exceeded, indicates a household has experienced catastrophic health spending.

Fig. 2 compares the distribution of catastrophic health spending by consumption quintile and method across countries. Using the budget share method (10 and 25% thresholds), the incidence of catastrophic spending was equally distributed across quintiles in some countries. In many countries, however, households in the richest quintile were more likely than households in the poorest quintile to experience financial hardship. The actual food spending and the partial normative food-spending methods produced similar distributions to the budget share methods, although in many countries there was a slightly higher incidence of catastrophic spending among the poorest quintile when using these methods than with the budget share method. The normative spending on food, housing and utilities method showed that the poorest quintile was most affected by catastrophic health spending in all countries.

**Fig. 3:**
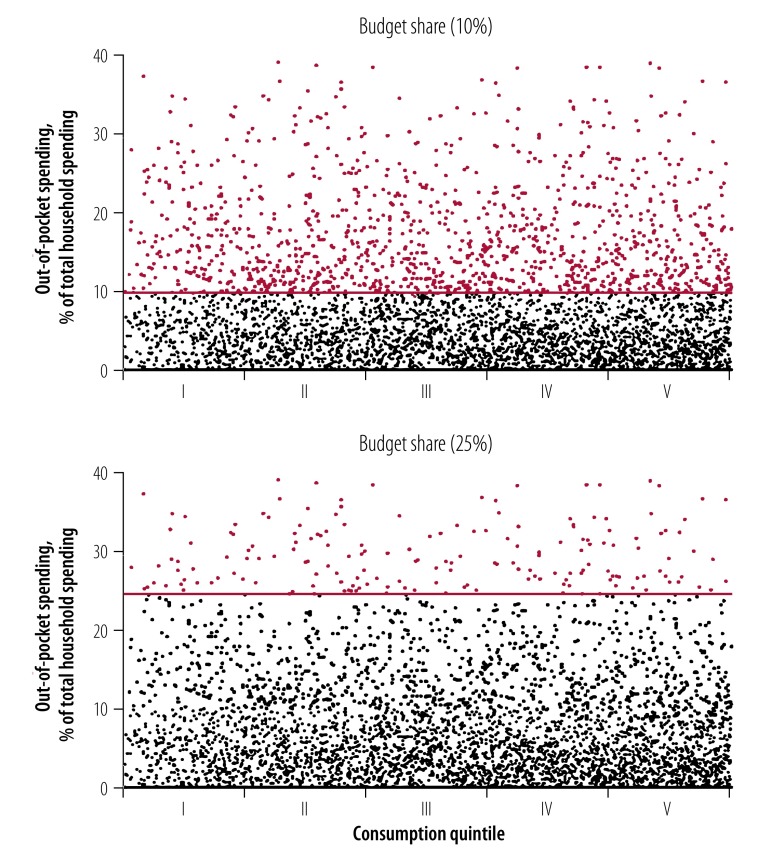
Out-of-pocket payments as a share of household expenditure among 6931 households in Lithuania in 2012

Notes: Households are sorted from left to right by per equivalent person consumption expenditure. Blue dots indicate out-of-pocket payments as a share of total household expenditure for a household that has experienced catastrophic spending using the budget share method at the respective threshold. Black dots indicate out-of-pocket payments as a share of total household expenditure for a household that has not experienced catastrophic spending. Horizontal lines are the thresholds for households to be counted as catastrophic spenders. Calculated using data from the 2012 Lithuanian household budget survey.

To better understand why the four methods produced different catastrophic health spending incidences and distributions across quintiles, we explored a single country’s household-level data in greater detail. Fig. 3 ranks households from the 2012 Lithuanian household budget survey by total household expenditure adjusted for household size and composition (i.e. from poor on the left to rich on the right). Figure 3 distinguished between households whose out-of-pocket payments as a share of total household expenditure were above and below the 10% and 25% thresholds used in the budget share method. We can see that there was not much variation in the out-of-pocket shares of total household consumption expenditure between rich and poor.

**Fig. 4:**
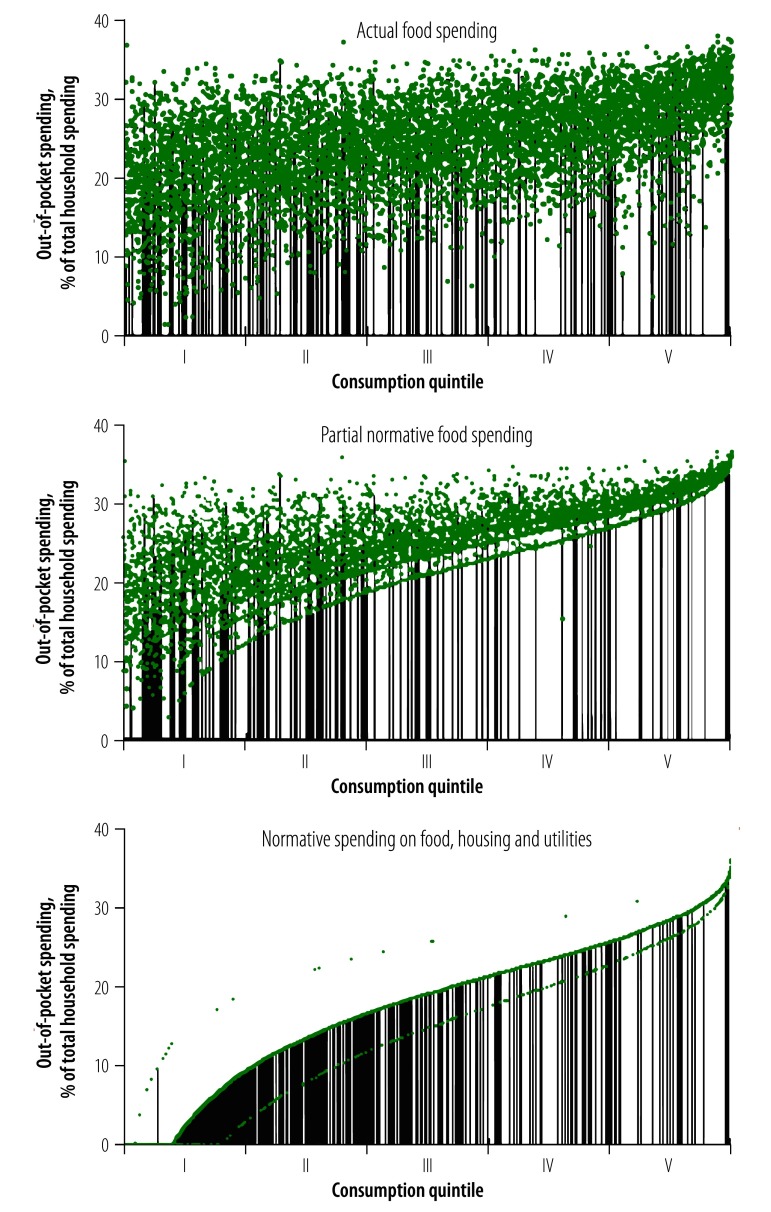
Share of total household expenditure that would have to be spent out-of-pocket to be counted as having catastrophic health spending among 6931 households in Lithuania in 2012, using different methods of calculation

Notes: Households are sorted from left to right by per equivalent person consumption expenditure. Each blue dot represents one household and indicates the effective threshold to be counted as having catastrophic spending. Black bars indicate households identified as catastrophic spenders. Calculated using data from the 2012 Lithuanian Household Budget Survey. The appearance of multiple curves in the panel showing the normative spending on food, housing and utilities method is because some households do not report any spending on utilities or rent, so the standard amount deducted from these households’ budgets excludes these items.

Fig. 4 shows the same Lithuanian households ranked by total household expenditure adjusted for household size and composition. The chart highlights the budget share each household would need to spend on health care to be counted as a catastrophic spender, using the three capacity-to-pay methods that is, the actual or effective threshold per household. The effective threshold for each household is represented by a single dot in all three panels, although in some instances the dots appear to form a curve or line. To make it easier to visualize the distribution of catastrophic spending, the vertical lines indicate households counted as catastrophic spenders. Here, we note that for the actual food and partial normative food-spending methods, there was considerable variation in the effective threshold, particularly among poorer households, so that households with similar levels of total household expenditure may be held to quite different standards to be counted as catastrophic spenders. For the normative spending on food, housing and utilities method, richer households must spend a progressively greater share of their budget to be counted as catastrophic spenders. 

**Fig. 5:**
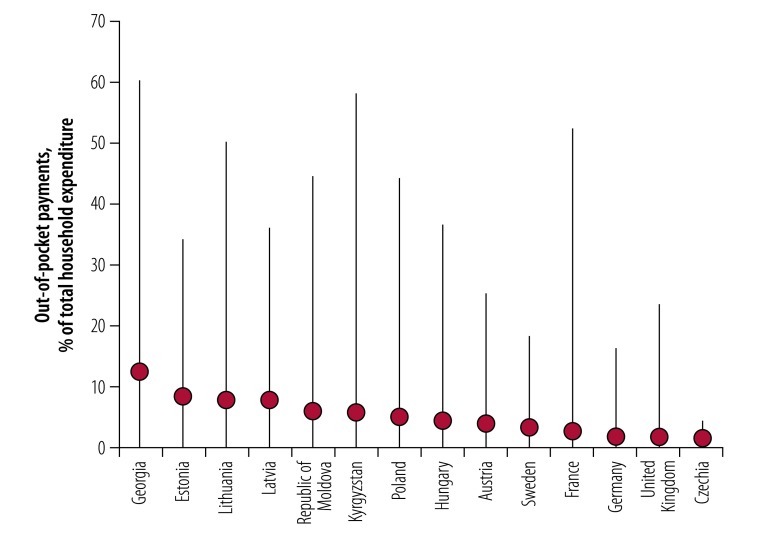
Out-of-pocket payments as a share of total household expenditure among households who are further impoverished by out-of-pocket payments in 14 European countries

Notes: Survey years and samples sizes are listed in the footnote to Fig. 1. Further impoverished households have total household expenditure below the normative amount of spending on food, housing and utilities. The circles represent mean values and the vertical lines show the interval between minimum and maximum values.

Lastly, unlike for the other three methods, the normative spending on food, housing and utilities method may result in some very poor households having negative capacity-to-pay. We refer to households whose total budget is below the standard amount for basic needs and who also incur any level of out-of-pocket payments as households who are further impoverished by out-of-pocket payments; all households further impoverished by out-of-pocket payments are considered to be catastrophic spenders. We found that across countries, the average household who was further impoverished spent between 1.4 and 12.0% of their budget out-of-pocket (Fig. 5). In 13 of the 14 countries the average household further impoverished by out-of-pocket payments spent less than the budget share that would be needed to be counted using the 10% SDG threshold (Fig. 5).

## Discussion

With the budget share method, once out-of-pocket health spending crosses a predefined budget share threshold, a household is considered to be a catastrophic spender. As noted in the example of Lithuania, there is not much visible variation in the out-of-pocket shares of total household expenditure between rich and poor. This explains why both rich and poor households have similar likelihoods of spending above or below the 10% or 25% thresholds. Global studies based on the budget share method have also found a higher incidence of catastrophic spending among the richest quintile.^3^^,^^7^

The budget share method is analogous to a flat income tax, which requires all households to pay the same share of their income in taxes. While this may seem fair to some people, it fails to acknowledge that poor people devote more of their resources to meeting basic needs than rich people. Poor people will therefore face greater financial pressure than rich households who spend the same budget share on health. Applying the same threshold to all households, regardless of wealth, overstates ability to pay among poor households, leading to an underestimation of financial hardship among the poor, especially at the higher threshold of 25%. It is also likely to understate ability to pay among rich households, leading to an overestimation of financial hardship among the rich, especially at the lower threshold of 10%.

A more equitable way of capturing financial hardship is to use an effective threshold that rises with household expenditure. One way to do this is to establish, for each household, an amount that is protected from out-of-pocket payments. This is analogous to an income tax system with a tax-free allowance, where a minimum level of income is untaxed for all households; the effective tax rate is no longer the same for all households, but rises with income.

Capacity-to-pay approaches attempt to achieve this when measuring catastrophic spending. However, their ability to produce an effective threshold that rises with household expenditure is achieved with varying degrees of success (as shown in Fig. 4), depending largely on how consistently they treat households.

The actual food-spending method determines each household’s capacity-to-pay by deducting actual spending on food from its budget. One can question whether food is an adequate proxy for basic needs. More importantly, actual spending on food reflects household preferences and other characteristics that may be linked to out-of-pocket payments. For example, if a household spends less on food, because it needs to spend more on health care, it would appear to have greater capacity-to-pay than a comparable household with higher spending on food who would be less likely to be counted as a catastrophic spender. Using the actual food-spending method there is considerable variation in the effective threshold among households with comparable total household expenditure per equivalent person. While rich households generally do need to spend a greater share of their budget on health care than very poor households to count as catastrophic spenders, there is substantial variability in the share of out-of-pocket spending needed to be counted as a catastrophic spender among poorer households towards the left of the panel.

The partial normative food-spending method addresses the limitations of the actual food-spending method by deducting a standard amount of food spending from each household’s budget. This standard amount is based on the average per equivalent person food spending of households whose food share of total household expenditure is between the 45th and 55th percentiles of the total sample and then adjusted to reflect household size and composition.^1^ This aims to arrive at a standard expenditure level representing basic needs. Since it is based on spending among households ranked by share of total household expenditure on food (rather than ranked by total household expenditure) it captures food spending among a random mix of both rich and poor households. Where households spend less on food than the standard amount, the method deducts actual spending on food to ensure no household is left with negative capacity-to-pay. This is why we refer to the method as being only partially normative. Households whose food spending is just above or just below the standard food amount are treated differently.

As noted previously, both of the food-based methods achieve very similar results in terms of overall incidence of catastrophic spending.^13^ The reason for this is that, in all the countries we have analysed, the majority of households report actual food spending levels below the standard amount and are therefore treated identically in both methods. This can be seen in Fig. 4, where many of the effective thresholds are the same in panels showing actual food spending method and partial normative food spending method, particularly among poorer households, and many of the same households are counted as catastrophic spenders. Consequently, both of the food-based methods are likely to overstate the ability to pay of poorer households relative to richer households. Nevertheless, in contrast to the actual food-spending method, the normative food-spending method has a well-defined effective threshold that rises with total household expenditure, although most obviously among richer households.

The normative spending on food, housing and utilities method builds on the partial normative food-spending method but differs in three ways. First, it considers housing (rent) and utilities (water, electricity, gas and other fuels) as representative of basic needs, in addition to food. Second, to determine the standard amount representing subsistence spending on food, rent and utilities, the method calculates average spending on these items among households between the 25th and 35th percentiles of the total sample ranked by total household expenditure per equivalent person (rather than ranked by food share, as with the partial normative food method). These households are assumed to be not so poor as to be potentially under-spending on basic needs, but also not too close to the median. Third, the method allows a household to have negative capacity-to-pay.^21^ A standard amount is deducted from all households that spend on food, rent and utilities, respectively. Households with budgets below the standard amount for basic needs are considered to be catastrophic spenders if they incur any out-of-pocket payments.

The panel with normative spending on food, housing and utilities method in Fig. 4 shows how deducting a standard amount from all households’ budgets leads to an effective threshold that is consistently lowest among the poor and rises evenly as household consumption expenditure rises. The result is a greater concentration of catastrophic spending among poor households than the other methods. In the normative food, housing and utilities method, the standard amount operates in exactly the same way as a tax-free allowance and can be justified for the same reason: it does not make sense from an equity or efficiency standpoint to tax very low incomes. Many health systems adopt a similar approach when exempting low-income households from out-of-pocket co-payments.

One may question the appropriateness of counting as a catastrophic spender, any household with a total budget below the standard amount for basic needs that incurs out-of-pocket payments. However, households with budgets below the standard amount are a very small share of the total population, well below official European Union poverty incidence rates.^22^ The majority typically do not incur any out-of-pocket payments, so are not counted as catastrophic spenders. Furthermore, among those who do incur out-of-pocket payments, becoming further impoverished, the amounts they spend out-of-pocket can be substantial, particularly given how poor they are (Fig. 5).

An important limitation of any analysis using household budget survey data is that surveys may differ in terms of reporting period and the level of detail of the information collected. Additionally, all expenditures, including formal and informal health payments, are self-reported by household members and subject to reporting biases. Lastly, household budget surveys do not typically collect information on health-care needs, making it impossible to assess whether households who do not report out-of-pocket payments have unmet need for health care.

A commitment to “leave no one behind” is at the heart of the SDG agenda. We found that the method selected for measuring catastrophic health spending for the SDGs was the most likely to underestimate financial hardship among poor people and overestimate hardship among rich people. This finding has important implications for monitoring progress towards UHC in the SDGs, and especially for highlighting inequalities within and across countries, which is central to the SDG agenda. The finding also has implications for the development of appropriate policy responses if policymakers receive misleading information about patterns of catastrophic health spending. Fortunately, other methods of measuring catastrophic health spending offer a way forward. Capacity-to-pay approaches are a direct response to the limitations of the budget share method. While each method attempts to address the limitations of earlier methods, the normative food, rent and utilities method consistently deducts a standardized amount from all households. It is therefore able to achieve an effective threshold that rises in line with household wealth. 
